# Supplementary material for: Mechanisms governing the pioneering and redistribution capabilities of the non-classical pioneer PU.1
Source: Nat Commun. 2020 Jan 21;11:402. doi: 10.1038/s41467-019-13960-2 (PMC6972792; doi:10.1038/s41467-019-13960-2)
Supplement: Supplementary file 7 — Source data [file 41467_2019_13960_MOESM7_ESM.zip › Source_Data/Figure6/Figure6B_MotifScanOutput/FLI1_mutPU.1/homerResults/motif2.similar.html]

motif2

## Information for motif2

T
G
A
C
A
C
T
G
A
C
T
G
C
T
G
A
G
C
T
A
C
T
A
G
A
G
C
T
C
T
A
G
  
Reverse Opposite:  

A
G
T
C
C
T
G
A
A
G
T
C
C
G
A
T
A
G
C
T
A
G
T
C
T
G
A
C
A
C
T
G
  

|  |  |
| --- | --- |
| p-value: | 1e-932 |
| log p-value: | -2.147e+03 |
| Information Content per bp: | 1.739 |
| Number of Target Sequences with motif | 3151.0 |
| Percentage of Target Sequences with motif | 30.71% |
| Number of Background Sequences with motif | 2122.6 |
| Percentage of Background Sequences with motif | 5.57% |
| Average Position of motif in Targets | 103.7 +/- 51.0bp |
| Average Position of motif in Background | 97.3 +/- 65.0bp |
| Strand Bias (log2 ratio + to - strand density) | -0.1 |
| Multiplicity (# of sites on avg that occur together) | 1.03 |
| Motif File: | file (matrix) reverse opposite |

### Similar de novo motifs found

|  |  |  |  |  |  |  |  |
| --- | --- | --- | --- | --- | --- | --- | --- |
| Rank | Match Score | Redundant Motif | P-value | log P-value | % of Targets | % of Background | Motif file |
| 1 | 0.897 | G T A C T G A C C T A G T A C G T G C A C G A T C T A G C G A T C A T G T C A G A T G C | 1e-728 | -1677.382201 | 24.39% | 4.32% | motif file (matrix) |
| 2 | 0.847 | T C A G G A T C T A G C C G T A A G T C C T G A G C A T T A G C T A G C A C G T A T C G G A T C | 1e-223 | -513.770435 | 11.38% | 3.01% | motif file (matrix) |
| 3 | 0.814 | A T G C G A C T G A C T G T C A A T G C A G T C C G T A A G T C C G A T A C G T A T G C A G T C G T A C G A T C | 1e-157 | -361.840901 | 6.01% | 1.14% | motif file (matrix) |
| 4 | 0.819 | C T G A T A G C T G C A A C T G A C T G T G C A A C G T A C T G C G A T A C T G C T A G A C T G T C A G C T G A | 1e-132 | -304.864105 | 3.42% | 0.33% | motif file (matrix) |
| 5 | 0.868 | G A T C G T A C C G T A A G T C A C G T G C A T A T G C A G T C A G C T G T A C A G C T G A C T | 1e-131 | -303.417857 | 3.79% | 0.46% | motif file (matrix) |
| 6 | 0.751 | T G C A T A G C G A C T C T G A T A G C G T C A T A G C G A C T A G C T G A T C G T A C | 1e-53 | -122.400692 | 3.28% | 1.00% | motif file (matrix) |
